# Supplementary material for: Decoding health disparities by gender, ethnicity and chronic diseases across three Latin American countries
Source: Nat Commun. 2026 Feb 27;17:3854. doi: 10.1038/s41467-025-67564-0 (PMC13121632; doi:10.1038/s41467-025-67564-0)
Supplement: Supplementary file 1 — Supplementary Information [file 41467_2025_67564_MOESM1_ESM.pdf]

# Decoding health disparities by gender, ethnicity and chronic diseases across three Latin American countries

## SUPPLEMENTARY INFORMATION

### Methodological Aspects of Health and Nutrition Surveys

### ENSANUT 2018 (Mexico)

- **Sampling Design**: A probabilistic, stratified, and cluster sampling method was employed. The observation unit was the household, while the sampling unit was the dwelling. The sampling frame was based on the National Institute of Statistics and Geography (INEGI) Master Sample of Dwellings, constructed from the 2010 Population and Housing Census.
- **Sample Size**: For the health component, 50,000 households were selected across the 32 federal entities. For the nutrition component, the sample consisted of 32,000 households.
- **Data Collection Methods**: Information was collected through face-to-face interviews in selected households. Specific questionnaires were applied to different age and gender groups to obtain detailed data on various health and nutrition aspects.
- **Weighting Procedures**: Expansion factors were calculated for each household and for each individual within the selected households. These factors allow the survey results to be representative at both national and state levels.

### ENSANUT 2018 (Ecuador)

- **Sampling Design**: A two-stage stratified probabilistic sampling method was used. In the first stage, a stratified sample of Primary Sampling Units (PSUs) was selected with probability proportional to size, based on the total number of occupied private dwellings. In the second stage, households within each PSU were randomly selected, with an average of 18 households per PSU.
- **Sample Size**: A total of 2,591 clusters and 46,638 households were surveyed nationwide, covering all 24 provinces of the country.
- **Data Collection Methods**: Data collection was conducted through face-to-face interviews. Specific forms were used for different population groups, including children under five, women aged 10-49, men aged 12 and older, and children aged 5-17.
- **Weighting Procedures**: Expansion factors were calculated in two or three stages, depending on the target population. The first stage included weighting for the PSUs, with adjustments for non-response when necessary. The second stage involved weighting for households within the PSUs, also with adjustments for non-response and ineligibility. If a third selection stage was required, an additional weighting factor was calculated for the selected individuals.

### PNS 2019 (Brazil)

- **Sampling Design**: The National Health Survey (PNS) 2019 utilized a complex probabilistic sampling design in three stages. The first stage involved the selection of census tracts, the second stage selected households within the tracts, and the third stage selected an individual aged 15 or older within the household. The sampling was stratified and weighted to ensure national representativeness.
- **Sample Size**: The survey covered over 100,000 households across Brazil, ensuring statistical robustness at national and regional levels.
- **Data Collection Methods**: Data was collected through face-to-face interviews, with structured questionnaires applied to obtain information on demographic, socioeconomic, and health indicators.
- **Weighting Procedures**: The survey applied weighting adjustments to account for selection probabilities and non-response. Expansion factors were calculated to ensure representativeness at

different geographical levels, following the methodology established by the Brazilian Institute of Geography and Statistics (IBGE).

**Table S1. Variables, description, available and source for each country**

| Countrie | Variables | Description | Sourse |
|---|---|---|---|
| | Water | Binary variable: 1 if the villa has piped water, 0 if it does not have piped water. | ENSANUT (2018) |
| | Sewage | Binary variable, where 1 represents that the house's drainage is connected to the public network, septic tank or septic tank (biodigestor) and 0 for a pipe that leads to a ravine or a storm drain, a pipe that leads to a river, lake or sea and no drainage | ENSANUT (2018) |
| | Garbage | Binary variable, where 1 represents that in the house the basura collects a truck or a cart, the lever in the public basure or the lever in a container or deposit, and 0 for the options of those who man the basura, enters the basura , the tyrant on the street, on a ravine or the tyrant on a river or the sea. | ENSANUT (2018) |
| Mexico | Education | Ordinal variable with the following categories: "No education", "Complete primary", "Complete secondary", "Higher". "Complete secondary" includes secondary education and bachelor's degree, "Higher" includes bachelor's degree and postgraduate degrees. | ENSANUT (2018) |
| | Occupation | Variable with categories: Formal employee, Casual employee, Unemployed, Retired. Formal employee is obtained by combining the question about whether you have worked in the last week and whether you have received medical insurance for that job. | ENSANUT (2018) |
| | Gender | Question with the categories about whether the person was a man or woman. | ENSANUT (2018) |
| | Age | Age in completed years | ENSANUT (2018) |
| | Race/Ethnicity | Variable with the following categories: "mixed " and "Indigenous". For indigenous people, we take into account the question about whether the person speaks some indigenous language. | ENSANUT (2018) |

| | | | |
|---|---|---|---|
| | Urban/Rural | Binary variable where 0 represents that the house is located in a rural location (<2,500 inhabitants) and 1 for urban (2500-99,999 inhabitants) or metropolitan (>100,000 inhabitants) | ENSANUT (2018) |
| | Cronical desease | Constructed variable of self-report of having ever been diagnosed by a health professional with any of the following diseases: diabetes, b) cardiovascular disease, c) kidney disease, d) cerebrovascular disease, and d) obesity | ENSANUT (2018) |
| | Water | binary variable; 1 if the villa is supplied with piped water from the public network, 0 for other sources such as a distribution car/tricycle, by the river/ shed/ acequia. | ENSANUT (2018) |
| | Sewage | Binary variable, where 1 represents that the house's exhaust and water supply is connected to the public network, 0 for the options of: septic well, underground well, streetlight and the alcantarillado is not excluded. | ENSANUT (2018) |
| | Garbage | Binary variable, where 1 represents that in the house the basura is eliminated through the municipal service, 0 for the other options such as the botan on the street/brake/river, the man, the interior or contract the service. | ENSANUT (2018) |
| Ecuador | Education | Ordinal variable with the following categories: "No education", "Complete primary", "Complete secondary", "Higher". "Complete secondary" includes secondary education and bachelor's degree, "Higher" includes bachelor's degree and postgraduate degrees. | ENSANUT (2018) |
| | Occupation | Variable with categories: Formal employee, Casual employee, Unemployed, Retired. | ENSANUT (2018) |
| | Gender | Question with the categories about whether the person was a man or woman. | ENSANUT (2018) |
| | Age | Age in completed years | ENSANUT (2018) |
| | Race/Ethnicity | Variable auto identification category: "Mestizo", "Indigenous", "Others", "Black". For the "other" category contains the mulato/montuvio and white categories. For the Black category, the "Afro-Ecuadorian/Afro-descendant" category was considered. | ENSANUT (2018) |

| | | | |
|---|---|---|---|
| | Urban/Rural | Binary variable where 0 represents that the house is located in a rural location (<2,500 inhabitants) and 1 for urban (>2500 inhabitants) | ENSANUT (2018) |
| | Cronical desease | Constructed variable of self-report of having suffered from any illness in the last 30 days, taking into account the following illnesses: a) high blood pressure, b) diabetes, c) obesity, d) cancer, e) arthritis and f) cardiovascular | |
| | Water | Binary variable; 1 if the villa has piped water from the public network, 0 if it does not have piped water | PNS (2019) |
| | Sewage | Binary variable, where 1 represents that the house from which drains the bathrooms and inodors through a general drainage system or storm drain, septic tank or rudimentary black well and 0 for Zanja<br>Direct to the river, lake or sea, Otro. | PNS (2019) |
| | Garbage | Binary variable, where 1 represents that in the house the base is collected directly by the cleaning service or collected in the cleaning service cube. For 0 it represents the villa where the base is built, dug in, taken out on a vacant lot or taken into a river, lake or sea. | PNS (2019) |
| Brasil | | | |
| | Education | Ordinal variable with the following categories: "No education", "Complete primary", "Complete secondary", "Higher". "Complete secondary" includes secondary education and bachelor's degree, "Higher" includes bachelor's degree and postgraduate degrees. | PNS (2019) |
| | Occupation | Variable with categories: Formal employee, Casual employee, Unemployed, Retired. | PNS (2019) |
| | Gender | Question with the categories about whether the person was a man or woman. | PNS (2019) |
| | Age | Age in completed years | PNS (2019) |
| | Race/Ethnicity | Variable auto identification category: "Mestizo", "Indigenous", "Others", "Black". For the "other" category contains the categories White, Yellow. For the Black category, the "Black" category is taken into account. For mestizos, I consider the "Brown" category. | PNS (2019) |

| | | |
|---|---|---|
| Urban/Rural | Binary variable where 0 represents that the house is located in a rural location (<2,500 inhabitants) and 1 for urban (>2500 inhabitants) | PNS (2019) |
| Cronical desease | Constructed variable of self-report of having ever been diagnosed by a health professional with any of the following diseases: diabetes, b) cardiovascular disease, c) kidney disease, d) cerebrovascular disease, and d) obesity | PNS (2019) |

**Table S2. AUC and importance of the country variable through AUC lost for each gender-ethnicity group.**

| Etnicity | Gender | AUC | Percentage of AUC lost of the country variable |
|---|---|---|---|
| Black | Men | 0.7634093 | 1.47 % |
| Mixed | Men | 0.8265871 | 6.59 % |
| Indigenous | Men | 0.8629965 | 5.55 % |
| Others | Men | 0.7896242 | 2.47 % |
| Mixed | Women | 0.8194094 | 5.54 % |
| Black | Women | 0.7841532 | 4.75 % |
| Indigenous | Women | 0.8378872 | 7.74 % |
| Others | Women | 0.7544719 | 2.71 % |

Note: The table shows the total area under the curve for each of the trained random forest models by ethnicity-gender group and the importance of the country variable in each group measured by the area under the curve lost if this variable is neutralized. Source data are provided as a Source Data file.

Figure S10 shows the importance of variables by gender and ethnicity for Mexico. For indigenous women, piped water and occupation were the most important variables for predicting chronic disease diagnoses. For women of mixed ethnicity, education was the most important variable. For men, education was the most important variable for predicting chronic disease diagnoses in mixed ethnicity, and for indigenous men, it was occupation. The model for mixed-ethnic men had a performance of AUC = .87, the model for indigenous women had an AUC = .88. The model for mixed-ethnic women had a performance of AUC = .85, and the model for indigenous women had a performance of AUC = .83.

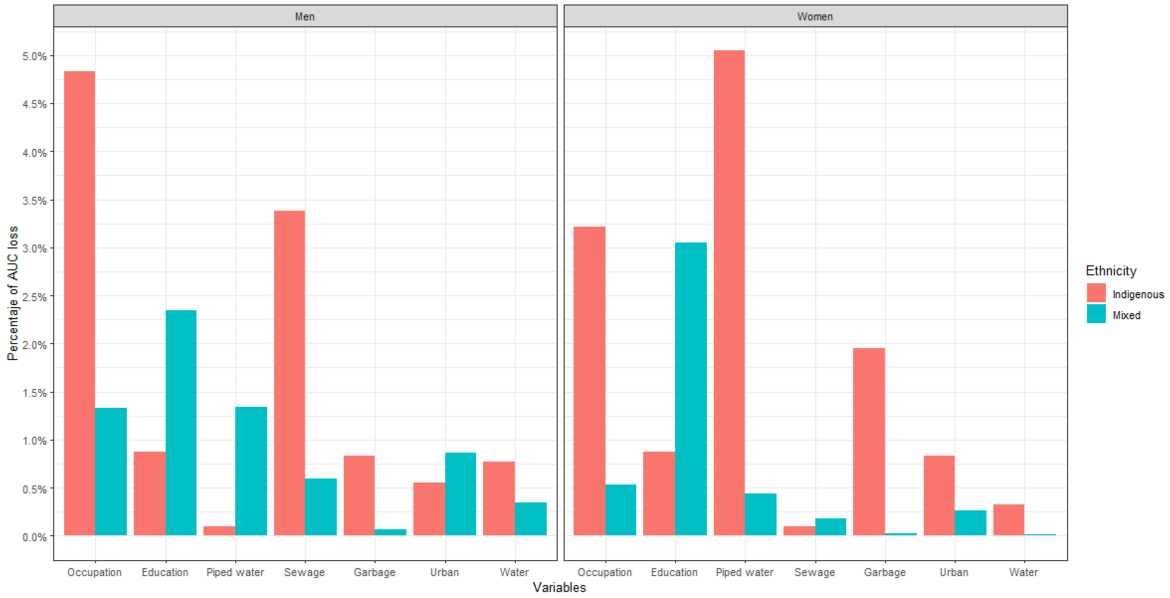


**Figure S1.** Importance of variables to predict the diagnosis of chronic diseases by the random forest model according to gender and ethnicity for the Mexican population. Source data are provided as a Source Data file.

Figure S2 shows the difference in the importance of variables by gender. For the indigenous population, piper water was the variable that differed most between men and women, being much more important for women. Meanwhile, for the mixed population, the piper water variable was also the most important, being more important for men. Source data are provided as a Source Data file.

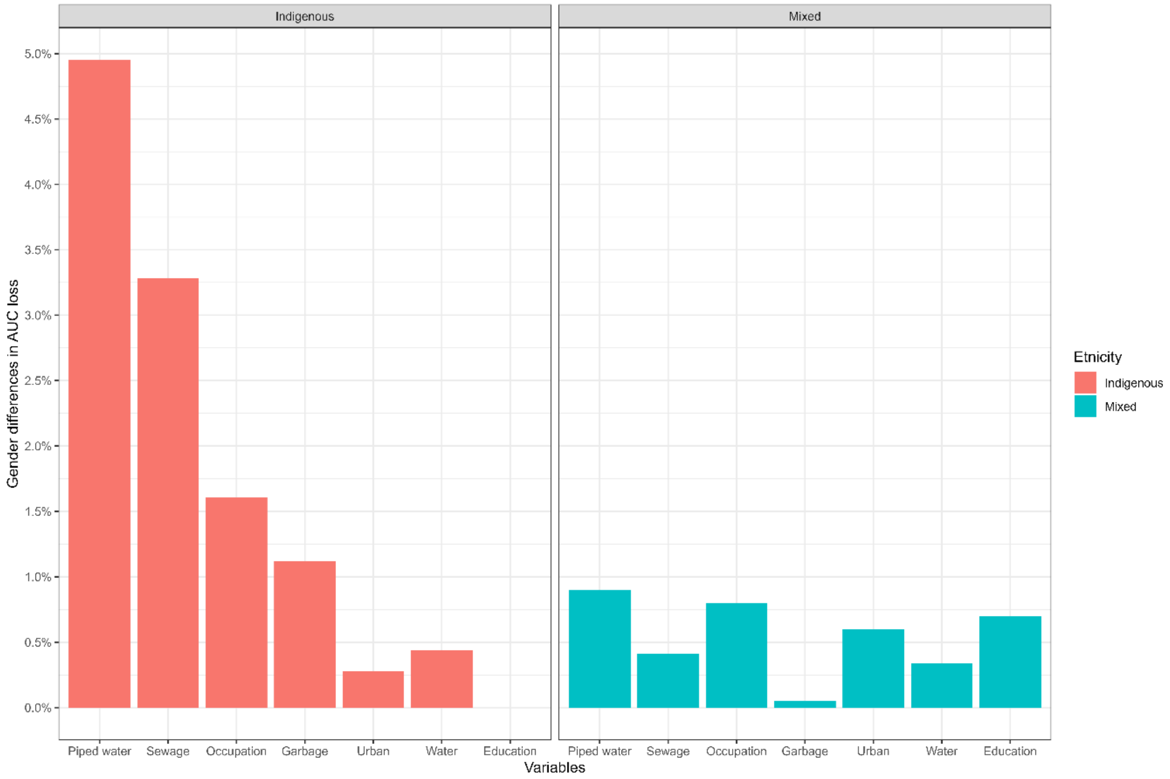

**Figure S2**. Difference in AUC lost between genders for each ethnicity group in Mexico. Source data are provided as a Source Data file.

Figure S3 shows the importance of variables in predicting chronic disease diagnoses in the Brazilian population. For women, occupation was the most important variable in almost all ethnic groups (except for the other ethnic group). For men, occupation was also the most important variable for all ethnic groups. The model for mixed-ethnic men had a performance of AUC = .83, the model for black men had an AUC = .83, the model for indigenous men had an AUC = .74 and the model for others men had AUC = .75. The model for mixed-ethnic women had a performance of AUC = .69, the model for black women had an AUC = 68., the model for indigenous women had an AUC =.67, and the model for others women had AUC = 71.

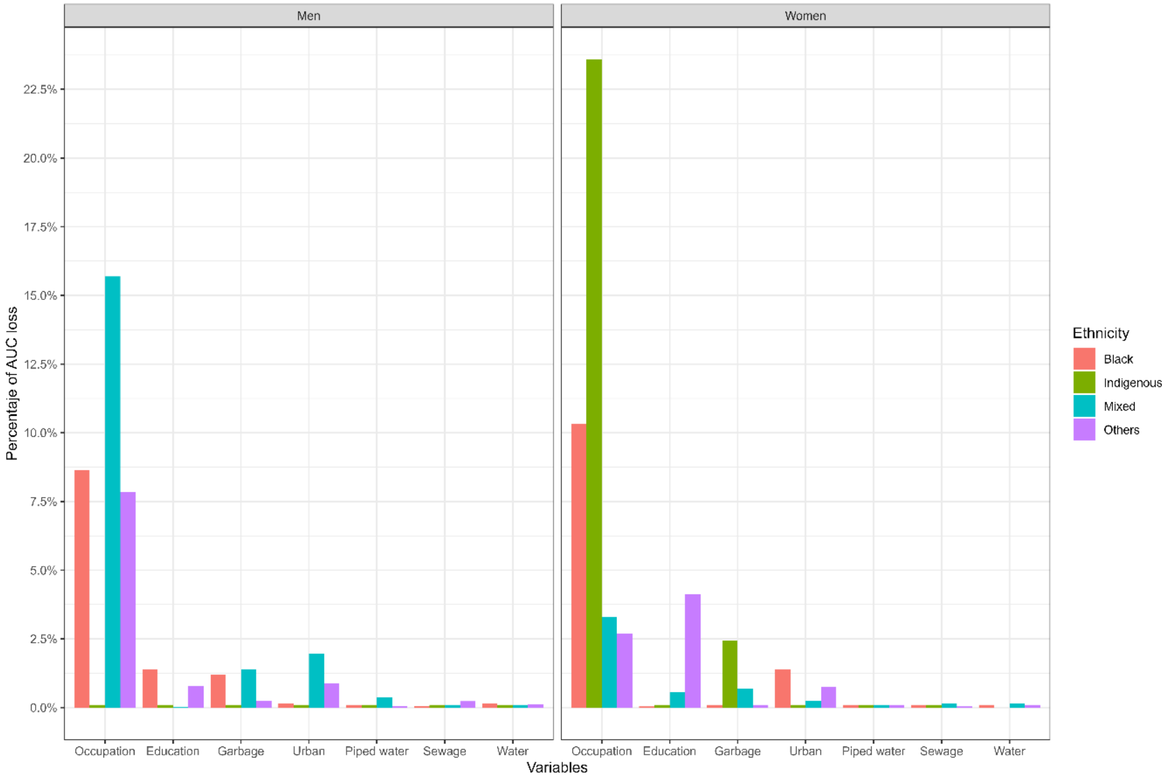


**Figure S3.** Importance of variables to predict the diagnosis of chronic diseases by gender and ethnic in Brazil. Source data are provided as a Source Data file.

Figure S4 shows the difference in missing AUC between genders for each ethnic group. For the Black population, the most important difference between genders was occupation, which was much more important for women than for men. Similarly, for the Indigenous population, occupation was the most important, but more so for women. For the mestizo population, occupation was the most important difference between genders, but more so for men. For the other group, occupation was also the variable that presented the greatest differences, being more important for men.

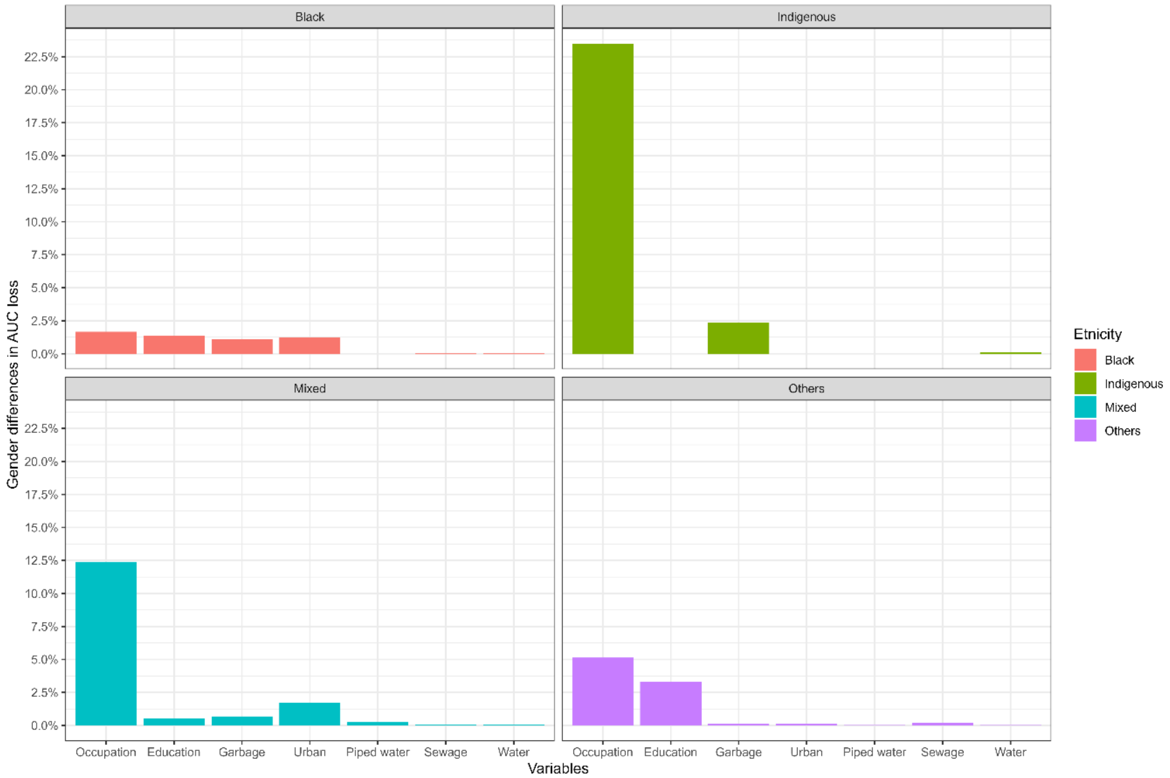


**Figure S4.** Difference in AUC lost between genders for each ethnicity group in Brazil. Source data are provided as a Source Data file.

Figure S5 shows the importance of variables in predicting chronic disease diagnoses in the Ecuadorian population. For women, education was the most important variable. For men, education, water, and living in an urban environment were the most important. The model for mixed-ethnic men had a performance of AUC = .76, the model for black men had an AUC = .65, the model for indigenous men had an AUC = .73 and the model for others men had AUC = .69. The model for mixed-ethnic women had a performance of AUC = .77, the model for black women had an AUC = .75., the model for indigenous women had an AUC =.71., and the model for others women had AUC = .73.

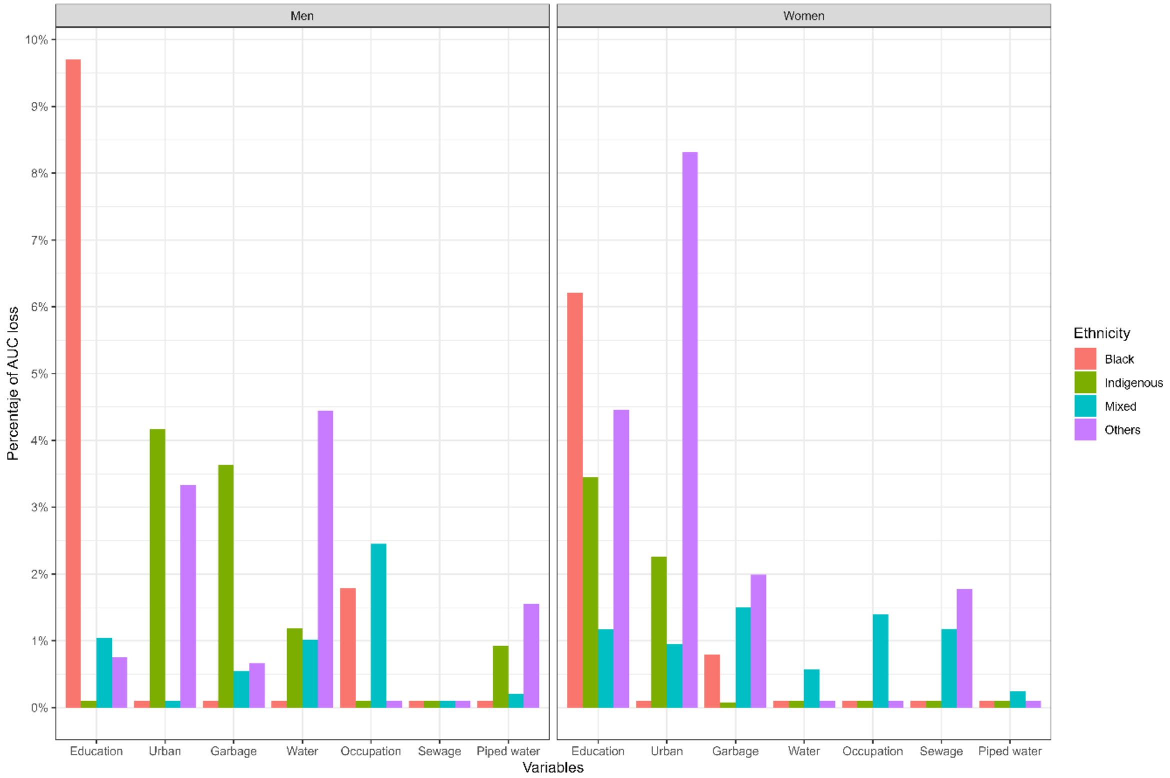


**Figure S5.** Importance of variables to predict the diagnosis of chronic diseases by gender and ethnic in Ecuador. Source data are provided as a Source Data file.

Figure S6 shows the difference in missing AUC by gender for each ethnicity in Ecuador. For the Black population, the main difference between genders was education, both of which were much more important for men than for women. For the Indigenous population, garbage was the most important variable, being more important for men. For the mestizo population, the differences between genders were smaller, with sewage being the most important. For the population of other ethnicities, urban potable water was the variable that presented the greatest differences, being more important for women than for men.

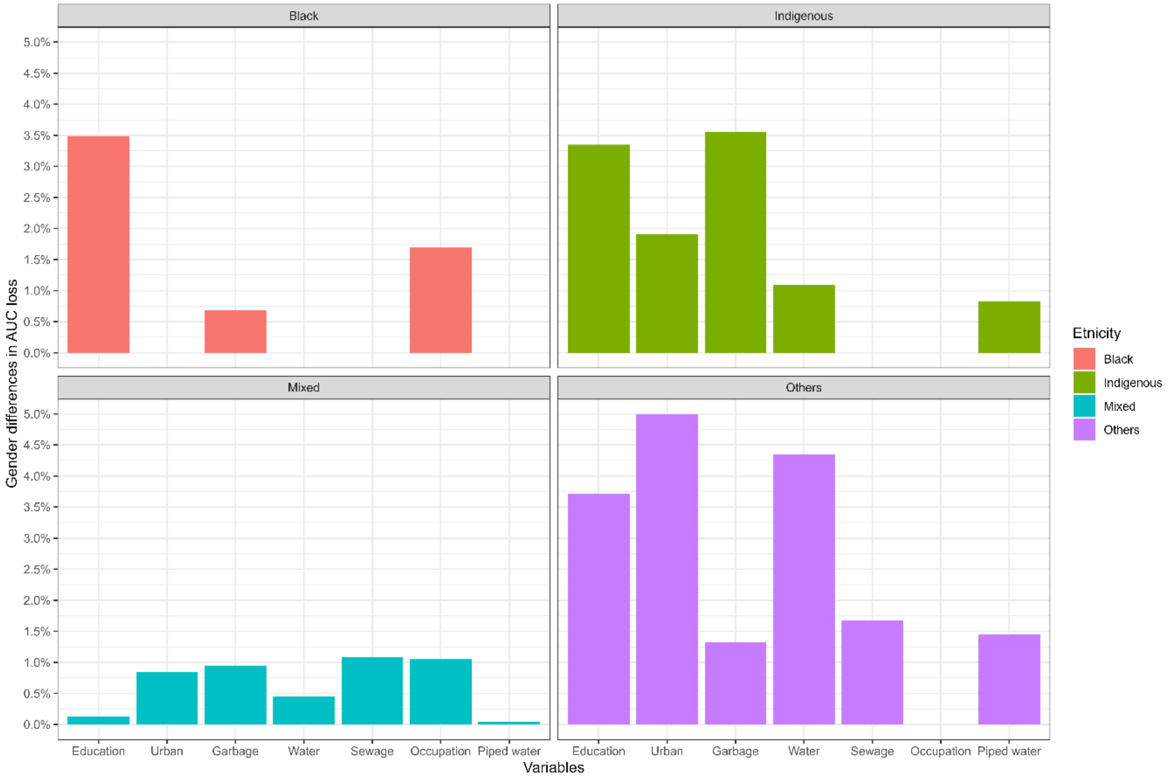


**Figure S6.** Difference in AUC lost between genders for each ethnicity group in Ecuador. Source data are provided as a Source Data file.

**Table S3. Descriptive analysis by gender and ethnicity group in Mexico**

| | Men | | | | Women | | | | |
|---|---|---|---|---|---|---|---|---|---|
| | Mixed | Black | Indigenous | Others | Mixed | Black | Indigenous | Others | p-value |
| **Dependent variable** | | | | | | | | | |
| Dx chronic disease | 0.11 | NA | 0.11 | NA | 0.14 | NA | 0.14 | NA | .00 |
| **Independent variables** | | | | | | | | | |
| Piped water | 0.77 | NA | 0.38 | NA | 0.94 | NA | 0.34 | NA | .00 |
| Water | 0.94 | NA | 0.81 | NA | 0.94 | NA | 0.81 | NA | .00 |
| Sewage | 0.96 | NA | 0.82 | NA | 0.96 | NA | 0.83 | NA | .00 |
| Garbage | 0.9 | NA | 0.58 | NA | 0.90 | NA | 0.57 | NA | .00 |
| Urban location | 0.80 | NA | 0.48 | NA | 0.80 | NA | 0.49 | NA | .00 |
| Occupation | | | | | | | | NA | |
| Informal employment | 0.46 | NA | 0.71 | NA | 0.28 | NA | 0.32 | NA | .00 |
| Formal employment | 0.34 | NA | 0.12 | NA | 0.19 | NA | 0.05 | NA | |
| Retiree | 0 | NA | 0 | NA | 0.0 | NA | 0.0 | NA | |
| Unemployed | 0.18 | NA | 0.16 | NA | 0.52 | NA | 0.61 | NA | |
| Education | | | | | | | | NA | |
| No Education | 0.28 | NA | 0.63 | NA | 0.32 | NA | 0.65 | NA | .00 |
| Education primary | 0.28 | NA | 0.21 | NA | 0.31 | NA | 0.23 | NA | |
| Education secondary | 0.20 | NA | 0.08 | NA | 0.18 | NA | 0.06 | NA | |
| Education Higher | 0.22 | NA | 0.05 | NA | 0.18 | NA | 0.04 | NA | |
| Age | 44.28 (16.52) | NA | 47.48 (16.9) | NA | 45.19 (16.75) | NA | 46.58 (17.42) | NA | .00 |

Note: All variables represent proportion, except for age where the mean and standard deviation are shown. One-sided chi-square tests are used to determine if there are statistical differences between genders, and two-sided Kolmogorov-Smirnov tests are used for age distribution. Source data are provided as a Source Data file.

**Table S4. Descriptive analysis by gender and ethnicity group in Brazil**

| | Men | | | | Women | | | | |
|---|---|---|---|---|---|---|---|---|---|
| | Mixed | Black | Indigenous | Others | Mixed | Black | Indigenous | Others | p-value |
| **Dependent variable** | | | | | | | | | |
| Dx chronic disease | 0.14 | 0.15 | 0.16 | 0.18 | 0.19 | 0.21 | 0.16 | 0.19 | .00 |
| **Independent variables** | | | | | | | | | |
| Piped water | 0.97 | 0.98 | 0.96 | 0.99 | 0.96 | 0.97 | 0.97 | 0.99 | .00 |
| Water | 0.97 | 0.99 | 0.98 | 0.99 | 0.97 | 0.99 | 0.98 | 0.99 | .00 |
| Sewage | 0.96 | 0.96 | 0.97 | 0.97 | 0.96 | 0.97 | 0.98 | 0.97 | .00 |
| Garbage | 0.90 | 0.92 | 0.95 | 0.95 | 0.91 | 0.95 | 0.96 | 0.96 | .00 |
| Urban location | 0.85 | 0.88 | 0.91 | 0.90 | 0.87 | 0.90 | 0.92 | 0.92 | .00 |
| Occupation | | | | | | | | | |
| Informal employment | 0.0 | 0.0 | 0.0 | 0.0 | 0.0 | 0.0 | 0.0 | 0.0 | .00 |
| Formal employment | 0.56 | 0.58 | 0.54 | 0.53 | 0.37 | 0.40 | 0.40 | 0.39 | |
| Retiree | 0.33 | 0.30 | 0.27 | 0.40 | 0.47 | 0.46 | 0.52 | 0.51 | |
| Unemployed | 0.10 | 0.10 | 0.18 | 0.05 | 0.14 | 0.12 | 0.07 | 0.09 | |
| Education | | | | | | | | | |
| No Education | 0.41 | 0.39 | 0.39 | 0.30 | 0.42 | 0.41 | 0.38 | 0.32 | .00 |
| Education primary | 0.16 | 0.15 | 0.16 | 0.12 | 0.11 | 0.12 | 0.23 | 0.09 | |
| Education secondary | 0.34 | 0.36 | 0.40 | 0.36 | 0.34 | 0.34 | 0.31 | 0.32 | |
| Education Higher | 0.08 | 0.08 | 0.03 | 0.20 | 0.11 | 0.11 | 0.06 | 0.25 | |
| Age | 47.02 (18.16) | 46.83 (17.73) | 45.27 (17.42) | 50.56 (18.42) | 50.1 (18.67) | 50.76 (18.67) | 51.64 (18.93) | 53.16 (18.79) | .00 |

Note: All variables represent proportion, except for age where the mean and standard deviation are shown. One-sided chi-square tests are used to determine if there are statistical differences between genders, and two-sided Kolmogorov-Smirnov tests are used for age distribution. Source data are provided as a Source Data file.

**Table S5. Descriptive analysis by gender and ethnicity group in Ecuador**

| | Men | | | | Women | | | | |
|---|---|---|---|---|---|---|---|---|---|
| | Mixed | Black | Indigenous | Others | Mixed | Black | Indigenous | Others | p-value |
| **Dependent variable** | | | | | | | | | |
| Dx chronic disease | 0.02 | 0.02 | 0.01 | 0.03 | 0.03 | 0.03 | 0.01 | 0.04 | .00 |
| **Independent variables** | | | | | | | | | |
| Piped water | 0.93 | 0.86 | 0.87 | 0.79 | 0.94 | 0.88 | 0.89 | 0.81 | .00 |
| Water | 0.84 | 0.77 | 0.57 | 0.61 | 0.85 | 0.80 | 0.59 | 0.62 | .00 |
| Sewage | 0.7 | 0.62 | 0.48 | 0.39 | 0.71 | 0.66 | 0.48 | 0.41 | .00 |
| Garbage | 0.89 | 0.89 | 0.71 | 0.66 | 0.90 | 0.89 | 0.71 | 0.68 | .00 |
| Urban location | 0.74 | 0.43 | 0.39 | 0.53 | 0.75 | 0.79 | 0.39 | 0.55 | .00 |
| Occupation | | | | | | | | | |
| Informal employment | 0.01 | 0.11 | 0.03 | 0.0 | 0.03 | 0.03 | 0.16 | 0.03 | .00 |
| Formal employment | 0.80 | 0.38 | 0.82 | 0.79 | 0.46 | 0.43 | 0.48 | 0.32 | |
| Retiree | 0.0 | 0.0 | 0.0 | 0.0 | 0.0 | 0.0 | 0.0 | 0.0 | |
| Unemployed | 0.17 | 0.37 | 0.14 | 0.2 | 0.49 | 0.53 | 0.35 | 0.64 | |
| Education | | | | | | | | | |
| No Education | 0.03 | 0.23 | 0.1 | 0.08 | 0.04 | 0.07 | 0.19 | 0.08 | .00 |
| Education primary | 0.38 | 0.49 | 0.49 | 0.51 | 0.37 | 0.41 | 0.49 | 0.51 | |
| Education secondary | 0.33 | 0.47 | 0.3 | 0.22 | 0.31 | 0.34 | 0.21 | 0.21 | |
| Education Higher | 0.24 | 0.33 | 0.09 | 0.17 | 0.26 | 0.16 | 0.08 | 0.18 | |
| Age | 42.52 (16.58) | 42 (15.67) | 41.33 (16.61) | 45.01 (17.83) | 43.08 (16.83) | 42.39 (16.67) | 42.55 (17.33) | 46.09 (17.51) | .00 |

Note: All variables represent proportion, except for age where the mean and standard deviation are shown. One-sided chi-square tests are used to determine if there are statistical differences between genders, and two-sided Kolmogorov-Smirnov tests are used for age distribution. Source data are provided as a Source Data file.
