# Supplementary material for: Decoding health disparities by gender, ethnicity and chronic diseases across three Latin American countries
Source: Nat Commun. 2026 Feb 27;17:3854. doi: 10.1038/s41467-025-67564-0 (PMC13121632; doi:10.1038/s41467-025-67564-0)
Supplement: Supplementary file 2 — Reporting Summary [file 41467_2025_67564_MOESM2_ESM.pdf]

Corresponding author(s): Carlos Chivardi
Last updated by author(s): Nov 18, 2025

Reporting Summary

Nature Portfolio wishes to improve the reproducibility of the work that we publish. This form provides structure for consistency and transparency in reporting. For further information on Nature Portfolio policies, see our Editorial Policies and the Editorial Policy Checklist.

Statistics

For all statistical analyses, confirm that the following items are present in the figure legend, table legend, main text, or Methods section.

n/a	Confirmed	
☐	☒	The exact sample size (*n*) for each experimental group/condition, given as a discrete number and unit of measurement
☐	☒	A statement on whether measurements were taken from distinct samples or whether the same sample was measured repeatedly
☐	☒	The statistical test(s) used AND whether they are one- or two-sided<br>*Only common tests should be described solely by name; describe more complex techniques in the Methods section.*
☐	☒	A description of all covariates tested
☒	☐	A description of any assumptions or corrections, such as tests of normality and adjustment for multiple comparisons
☐	☒	A full description of the statistical parameters including central tendency (e.g. means) or other basic estimates (e.g. regression coefficient) AND variation (e.g. standard deviation) or associated estimates of uncertainty (e.g. confidence intervals)
☐	☒	For null hypothesis testing, the test statistic (e.g. *F*, *t*, *r*) with confidence intervals, effect sizes, degrees of freedom and *P* value noted<br>*Give P values as exact values whenever suitable.*
☒	☐	For Bayesian analysis, information on the choice of priors and Markov chain Monte Carlo settings
☒	☐	For hierarchical and complex designs, identification of the appropriate level for tests and full reporting of outcomes
☒	☐	Estimates of effect sizes (e.g. Cohen's *d*, Pearson's *r*), indicating how they were calculated

Our web collection on statistics for biologists contains articles on many of the points above.

Software and code

Policy information about availability of computer code

Data collection	used individual-level, cross-sectional data from national health surveys in Mexico, Brazil, and Ecuador. Data sources included ENSANUT 2018 (Mexico), ENSANUT 2018 (Ecuador), and PNS 2019 (Brazil). Survey weights were applied to estimate the total adult population (20+ years), totaling around 97 million people across the three countries. The actual unweighted sample sizes were 42,068 for Mexico, 96,111 for Ecuador, and 40,088 for Brazil, distinguishing between raw observations and population-level estimates.
Data analysis	We carried out all the analyzes with the R 4.3 software in cloud computing using Google Colab.

For manuscripts utilizing custom algorithms or software that are central to the research but not yet described in published literature, software must be made available to editors and reviewers. We strongly encourage code deposition in a community repository (e.g. GitHub). See the Nature Portfolio guidelines for submitting code & software for further information.

Data

Policy information about availability of data

All manuscripts must include a data availability statement. This statement should provide the following information, where applicable:

- Accession codes, unique identifiers, or web links for publicly available datasets
- A description of any restrictions on data availability
- For clinical datasets or third party data, please ensure that the statement adheres to our policy

The data used in this study are publicly available from national health surveys conducted in Mexico, Brazil, and Ecuador. All datasets can be accessed through the respective government or institutional websites. No additional restrictions apply regarding data access or use.

Research involving human participants, their data, or biological material

Policy information about studies with human participants or human data. See also policy information about sex, gender (identity/presentation), and sexual orientation and race, ethnicity and racism.

Reporting on sex and gender	This study is based on the analysis of secondary data obtained from publicly accessible, nationally representative health surveys. The authors had no direct contact with human participants, nor did they collect biological samples or primary data. The datasets used were anonymized and de-identified before access. Therefore, ethical approval and participant consent were not required. The data source for Mexico was ENSANUT 2018 (Mexico), for Ecuador ENSANUT 2018 (Ecuador), and for Brazil PNS 2019 (Brazil).
Reporting on race, ethnicity, or other socially relevant groupings	This study is based on the analysis of secondary data obtained from publicly accessible, nationally representative health surveys. The authors had no direct contact with human participants, nor did they collect biological samples or primary data. The datasets used were anonymized and de-identified before access. Therefore, ethical approval and participant consent were not required. The data source for Mexico was ENSANUT 2018 (Mexico), for Ecuador ENSANUT 2018 (Ecuador), and for Brazil PNS 2019 (Brazil).
Population characteristics	This study is based on the analysis of secondary data obtained from publicly accessible, nationally representative health surveys. The authors had no direct contact with human participants, nor did they collect biological samples or primary data. The datasets used were anonymized and de-identified before access. Therefore, ethical approval and participant consent were not required. The data source for Mexico was ENSANUT 2018 (Mexico), for Ecuador ENSANUT 2018 (Ecuador), and for Brazil PNS 2019 (Brazil).
Recruitment	This study is based on the analysis of secondary data obtained from publicly accessible, nationally representative health surveys. The authors had no direct contact with human participants, nor did they collect biological samples or primary data. The datasets used were anonymized and de-identified before access. Therefore, ethical approval and participant consent were not required. The data source for Mexico was ENSANUT 2018 (Mexico), for Ecuador ENSANUT 2018 (Ecuador), and for Brazil PNS 2019 (Brazil).
Ethics oversight	This study is based on the analysis of secondary data obtained from publicly accessible, nationally representative health surveys. The authors had no direct contact with human participants, nor did they collect biological samples or primary data. The datasets used were anonymized and de-identified before access. Therefore, ethical approval and participant consent were not required. The data source for Mexico was ENSANUT 2018 (Mexico), for Ecuador ENSANUT 2018 (Ecuador), and for Brazil PNS 2019 (Brazil).

Note that full information on the approval of the study protocol must also be provided in the manuscript.

Field-specific reporting

Please select the one below that is the best fit for your research. If you are not sure, read the appropriate sections before making your selection.

☐ Life sciences ☒ Behavioural & social sciences ☐ Ecological, evolutionary & environmental sciences

For a reference copy of the document with all sections, see nature.com/documents/nr-reporting-summary-flat.pdf

Behavioural & social sciences study design

All studies must disclose on these points even when the disclosure is negative.

Study description	We conducted a multi-country, individual-level, cross-sectional quantitative study utilizing secondary data from large national health surveys in Brazil, Mexico, and Ecuador. The study employed machine learning techniques (random forest models) to analyze associations between social determinants (education, occupation, access to services) and the diagnosis of chronic diseases across gender and ethnic groups.
Research sample	The research sample included adult individuals (20+ years) from three national health surveys: ENSANUT 2018 (Mexico), ENSANUT 2018 (Ecuador), and PNS 2019 (Brazil). The unweighted sample sizes were 42,068 for Mexico, 96,111 for Ecuador, and 40,088 for

	Brazil. The surveys are nationally representative, capturing relevant demographic and socioeconomic characteristics, including gender, ethnicity, occupation, education, and access to services.
Sampling strategy	The study utilized pre-existing, nationally representative datasets where participants were selected through complex, stratified, multistage sampling methods as detailed in each country's survey methodology. For computational efficiency in the machine learning analysis, we randomly sampled 170,000 individuals from each gender/ethnic group. No additional sampling was conducted by the authors, and no sample size calculation was required beyond the national surveys' design.
Data collection	The datasets are publicly available, anonymized, and collected following each country's ethical standards.
Timing	As the study uses secondary, anonymized data from completed national surveys, there was no data collection.
Data exclusions	No individual-level data were excluded beyond the design of the original national health surveys. Variables were harmonized to ensure comparability across countries. No further exclusions were applied during the analysis.
Non-participation	As the study uses secondary, anonymized data from completed national surveys, there was no direct recruitment by the researchers, and therefore, no dropouts or non-participation in the study process.
Randomization	No applicable.

Reporting for specific materials, systems and methods

We require information from authors about some types of materials, experimental systems and methods used in many studies. Here, indicate whether each material, system or method listed is relevant to your study. If you are not sure if a list item applies to your research, read the appropriate section before selecting a response.

Materials & experimental systems

n/a	Involved in the study
☒	☐ Antibodies
☒	☐ Eukaryotic cell lines
☒	☐ Palaeontology and archaeology
☒	☐ Animals and other organisms
☒	☐ Clinical data
☒	☐ Dual use research of concern
☒	☐ Plants

Methods

n/a	Involved in the study
☒	☐ ChIP-seq
☒	☐ Flow cytometry
☒	☐ MRI-based neuroimaging

Plants

Seed stocks	No applicable
Novel plant genotypes	No applicable
Authentication	No applicable
